# Verrucous antral gastritis in relation to *Helicobacter pylori* infection, nutrition, and gastric atrophy

**DOI:** 10.1093/gastro/goz057

**Published:** 2019-10-30

**Authors:** Naoko Tsuji, Yasuko Umehara, Mamoru Takenaka, Yasunori Minami, Tomohiro Watanabe, Naoshi Nishida, Masatoshi Kudo

**Affiliations:** Department of Gastroenterology and Hepatology, Kindai University Faculty of Medicine, Osaka-Sayama, Japan

**Keywords:** verrucous gastritis, chromoendoscopy, BMI, *Helicobacter pylori;* eradication, gastric atrophy

## Abstract

**Background:**

There have been few studies in the English literature regarding verrucous gastritis (VG). The present study investigated the clinical and endoscopic features of verrucous antral gastritis, especially focusing on *Helicobacter pylori* infection, nutrition, and gastric atrophy.

**Methods:**

We performed a retrospective study of patients who underwent routine endoscopy with indigo carmine chromoendoscopy and a comparative study was conducted between VG-positive and VG-negative groups. VG was subdivided into classical and numerous types based on the number and distribution of verrucous lesions. Demographic, clinical, and endoscopic data including body mass index (BMI), serum albumin and cholesterol, gastric atrophy, reflux oesophagitis, Barrett’s oesophagus, and *H. pylori* status were collected. Univariate and multivariable analyses were performed to identify factors associated with VG.

**Results:**

We analysed the data of 621 patients undergoing routine endoscopy and found that VG (*n *=* *352) was significantly associated with increased BMI (1.12 [1.05–1.18], *P *<* *0.01), reflux esophagitis (1.96 [1.10–3.28], *P *<* *0.01), and *H. pylori* negativity with or without a history of eradication (9.94 [6.00–16.47] and 6.12 [3.51–10.68], *P *<* *0.001, respectively). Numerous-type (*n *=* *163) VG was associated with both closed- and open-type gastric atrophy (9.9 [4.04–21.37] and 8.10 [3.41–19.24], *P *<* *0.001, respectively). There were no statistical differences between groups regarding age, sex, total cholesterol, albumin, and bile-colored gastric juice.

**Conclusions:**

Verrucous antral gastritis was related to increased BMI, reflux esophagitis, and *H. pylori* negativity. Numerous-type verrucous lesions were associated with gastric atrophy. These indicate that VG may be a physiological phenomenon due to high gastric acidity, mechanical overload, and vulnerability of background mucosa.

## Introduction

Verrucous gastritis (VG) demonstrates multiple small flat or tiny elevated lesions with or without central depressions or erosions that involve the antrum. Endoscopically, this condition was already well known before the era of *Helicobacter pylori*, but its etiology and significance were not clear. The lesions are usually multiple and arranged linearly on the longitudinal fold of the distal antrum, converging towards the pylorus ring. Although it is termed gastritis, it is uncertain whether it is really an inflammation or illness. Because of its benign nature, this condition has not been studied in detail.

Few studies in the English literature have focused on VG. An association with duodenal and gastric ulceration and a male predominance were reported in the 1970s [1, 2]. In 2010, Yamamoto *et al.* [3] reported an increasing risk of endoscopic erosive gastritis in adults with a low serum adiponectin level and high body mass index (BMI). Their study population was sufficiently large (*n *=* *2,400); however, the status of *H. pylori* infection was unknown. In 2015, Kim *et al.* [4] reported that increased eating speed, high BMI, and male sex were risk factors for endoscopic erosive gastritis in 10,893 adults in a general health check-up. They also evaluated *H. pylori* status in some of the study population and concluded that the relation was unclear.

In recent years, obesity has been reported as a risk factor for some types of gastric cancer and erosive gastritis in Western countries [5, 6]; contrary to our previous idea that VG has a benign nature, recent reports suggested a link between VG and gastric cancer [7, 8].

The *H. pylori*-infection rate is high in the Japanese population and the National Health Insurance started covering two eradication regimens for all *H. pylori*-positive patients in 2013. Consequently, there has been an increased opportunity to perform endoscopy on patients after *H. pylori* eradication and we have occasionally found numerous verrucous elevations in the antrum of such patients. These elevations also lined the longitudinal folds but were occasionally crowded towards the oral antrum.

The present study aimed to investigate the clinical and endoscopic features of VG, particularly focusing on *H. pylori* infection, background gastric mucosa, and BMI with other nutritional status and analysed its relationship with gastric cancer.

## Patients and methods

### Patients

We reviewed the data of 1,250 consecutive patients who underwent routine esophagogastroduodenoscopy at Kindai University Faculty of Medicine (Osaka, Japan) between July and December 2015. We selected patients who underwent chromoendoscopy with 0.2% indigo carmine in the antrum and whose *H. pylori* statuses were ascertained via culture. Patients with advanced gastric cancer, those undergoing surgical gastrectomy, and those with insufficient endoscopic images were excluded. Medical and laboratory data at the same period of endoscopy were also collected by reviewing clinical records. To evaluate the nutritious states, heights and weights were collected to calculate the BMI. Laboratory data of serum total cholesterol and albumin, which were measured as a standard clinical practice just before endoscopy, were also collected. Follow-up data until the end of 2018 were also collected to investigate the occurrence of gastric cancer after the endoscopy period.

The study was conducted in accordance with the principles of the Helsinki Declaration and was approved by the local ethics committee of Kindai University Faculty of Medicine (IRB number: 28–077). Informed consent was waived because of the retrospective nature of this study.

### Evaluation of *H. pylori* infection

The *H. pylori*-infection status was confirmed via a culture method as a clinical practice and not specifically for this study. The history of *H. pylori* eradication in the clinical records was also reviewed. The *H. pylori* status was classified into three categories: positive, negative without a history of eradication, and negative after eradication at the time of endoscopy.

### Endoscopic assessments and definitions

**Figure 1.**
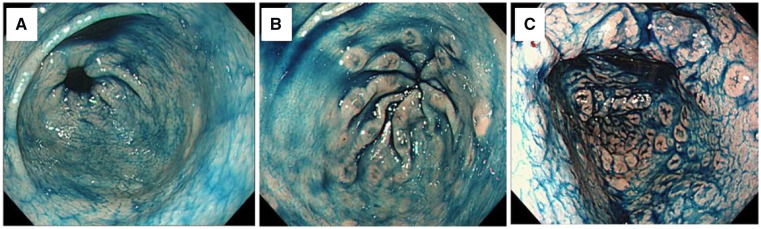
Endoscopic classification of verrucous gastritis (VG) assessed with indigo carmine dye spray. VG is classified into the VG-negative group (fewer than five verrucous lesions) (A) and the VG-positive group (at least five verrucous lesions). The VG-positive group is subclassified into the classical type (verrucous lesions were localized in the terminal antrum) (B) and the numerous type (number and/or distribution of verrucous lesions were large) (C).

Endoscopic images were reviewed and we defined VG as the presence of more than five verrucous lesions in the antrum identified by chromoendoscopic images. Patients with fewer than five verrucous lesions were included into the non-VG group, whereas those with at least five verrucous lesions were included in the VG group, which was subdivided into classical-type VG (classical VG, i.e. verrucous lesions were localized in the distal antrum and arranged along the longitudinal fold converging towards the pylorus ring) and numerous-type VG (numerous VG, i.e. numerous verrucous lesions that were occasionally not over the longitudinal fold and/or spread towards the oral antrum) (Figure 1A–C). Gastric mucosal atrophy was classified according to the Kimura–Takemoto classification as C-1, C-2, or C-3 (closed type) and as O-1, O-2, or O-3 (open type) [9]. In addition, patients without an atrophic border were classified as having no atrophy (C-0). Barrett’s esophagus, including ultrashort Barrett’s esophagus, and reflux esophagitis, including minimal change, were also evaluated. We also evaluated the presence or absence of bile-colored gastric juice in the gastric fundus.

### Statistical analyses

Continuous variables are presented as median and interquartile range, because the data did not show a normal distribution according to the Kolmogorov–Smirnov test. Categorical variables are displayed as frequencies and percentages. The frequencies of various characteristics were compared between the non-VG and VG groups, and between the classical VG and numerous VG groups. Statistical analysis was performed using the chi-squared or Fisher’s exact test for categorical data and the Mann–Whitney *U* test for continuous data. A multivariate logistic-regression model was applied to test which variables were independently associated with verrucous antral gastritis. SPSS version 24.0 (IBM Corp., Armonk, NY, USA) was used for the analysis and a *P*-value <0.05 was considered statistically significant.

## Results

### Clinical and endoscopic characteristics of patients with and without VG

#### Univariate analysis

A total of 621 subjects were included in this study (non-VG group, *n *=* *269; VG group, *n *=* *352). In the non-VG group, 165 patients had no verrucous lesions and 104 patients had one to four verrucous lesions. There were no significant differences between the two groups with respect to clinical background (i.e. age and sex distributions, total cholesterol, and albumin level). Only BMI was significantly higher in the VG group than in the non-VG group (22.6 vs 21.8 kg/m^2^, *P *<* *0.01).

Regarding endoscopic features, there was a significant difference in the degree of gastric mucosal atrophy between the two groups (*P *=* *0.04). The proportions of patients with reflux esophagitis (25.2%) and Barrett’s esophagus (22.7%) were significantly higher in the VG group than in the non-VG group (8.9% and 14.8%, *P *<* *0.01 and *P *=* *0.01, respectively), although most of the reflux esophagitis cases showed minimal change and the Barrett’s esophagitis cases were the ultrashort type. With respect to *H. pylori* status, 51.3% of non-VG patients and 11.9% of VG patients were *H. pylori*-positive (*P *<* *0.01). The frequency of bile-colored gastric juice was not significantly different between the two groups (Table 1).

**Table 1.** Demographic and endoscopic characteristics of individuals with and without VG

	Non-VG group	VG group	
Characteristic	(*n* = 269)	(*n* = 352)	*P*-value
Age (years), median [IQR]	69 [63–75]	70 [64–74]	0.73
Male gender, *n* (%)	106 (39.4)	162 (46.0)	0.1
BMI (kg/m^2^), median [IQR]	21.8 [19.7–23.8]	22.6 [20.7–25.0]	<0.01
Total cholesterol (mmol/L), median [IQR]	5.28 [4.66–5.98]	5.15 [4.58–5.75]	0.18
Albumin (g/L), median [IQR]	43.0 [40.0–45.0]	43.0 [40.0–45.0]	0.96
Gastric mucosal atrophy, *n* (%)			
No atrophy	46 (17.1)	84 (23.8)	
Closed-type atrophy	109 (40.5)	149 (42.3)	0.04
Open-type atrophy	114 (42.4)	119 (33.8)	
Reflux esophagitis, *n* (%)	24 (8.9)	89 (25.2)	<0.01
Barrett’s esophagus, *n* (%)	40 (14.8)	80 (22.7)	0.01
Bile-colored gastric juice, *n* (%)	43 (16.0)	53 (15.0)	0.75
*H. pylori* infection, *n* (%)			
Positive	138 (51.3)	42 (11.9)	
Negative			
Without a history of eradication	77 (28.6)	153 (43.4)	
After eradication	54 (20.0)	157 (44.6)	<0.001

IQR, interquartile range; BMI, body mass index; VG, verrucous gastritis; non-VG, without verrucous gastritis.

#### Multivariate analysis

The multivariate analysis revealed that BMI (odds ratio [OR] 1.12, 95% confidence interval [CI] 1.05–1.18, *P *<* *0.01) and reflux esophagitis (OR 1.96, 95% CI 1.10–3.28, *P *<* *0.01) were significantly associated with VG status. *H. pylori* negativity was independently associated with VG regardless of the history of eradication (negative without a history of eradication/positive, OR 6.12, 95% CI 3.51–10.68, *P *<* *0.01; negative after eradication/positive, OR 9.94, 95% CI 6.60–16.47, *P *<* *0.01) (Table 2).

**Table 2.** Multivariate logistic-regression analysis for VG group compared with non-VG group

Characteristic	OR	95% CI	*P*-value
Age (years)	1.01	0.99–1.03	0.4
Male gender	1.07	0.71–1.60	0.75
BMI (kg/m^2^)	1.12	1.05–1.18	<0.01
Total cholesterol (mmol/L)	0.99	0.99–1.00	0.06
Albumin (g/L)	1.04	0.65–1.67	0.88
Gastric mucosal atrophy			
No atrophy	1.00		
Closed-type atrophy	1.03	0.56–1.88	0.93
Open-type atrophy	0.84	0.45–1.58	0.59
Reflux esophagitis	1.96	1.10–3.28	0.02
Barrett’s esophagus	1.32	0.80–2.18	0.27
Bile-colored gastric juice	0.77	0.46–1.28	0.31
*H. pylori* infection			
Positive	1.00		
Negative			
Without a history of eradication	6.12	3.51–10.68	<0.01
After eradication	9.94	6.00–16.47	<0.01

BMI, body mass index; VG, verrucous gastritis; non-VG, without verrucous gastritis.

### Comparison between classical- and numerous-type VG

#### Univariate analysis

Of 352 VG patients, 189 and 163 patients were further classified into classical and numerous VG, respectively. There were no significant differences between the two groups with respect to clinical and nutritional backgrounds, including BMI. The proportion of patients with reflux esophagitis, Barrett’s esophagus, and bile-colored gastric juice did not significantly differ between both groups. Regarding gastric atrophy, 37.5% of patients with classical VG had no atrophy. However, only 7.9% of patients with numerous VG had no atrophy, whereas the rest had some degree of atrophy (*P *<* *0.01).

The *H. pylori* status was significantly different between the two groups. The *H. pylori*-positive rate was low in both groups, being only 3.6% in patients with numerous VG. Furthermore, 63.8% of patients with numerous VG were *H. pylori*-negative after eradication therapy (*P *<* *0.01) (Table 3).

**Table 3.** Comparison of patient characteristics between classical- and numerous-type verrucous gastritis patients

	Classical VG	Numerous VG	
Characteristic	(*n* = 189)	(*n* = 163)	*P*-value
Age (years), median [IQR]	69 [62–75]	70 [66–74]	0.37
Male gender, *n* (% )	79 (41.7)	83 (50.9)	0.09
BMI (kg/m^2^), median [IQR]	22.3 [20.4–24.7]	22.9 [21.1–25.3]	0.13
Total cholesterol (mmol/L), median [IQR]	5.02 [4.53–5.75]	5.23 [4.71–5.80]	0.21
Albumin (g/L), median [IQR]	43.0 [40.0–45.0]	43.0 [40.0–45.0]	0.44
Gastric mucosal atrophy, *n* (%)			
No atrophy	71 (37.5)	13 (7.9)	
Closed-type atrophy	61 (32.2)	88 (53.9)	<0.01
Open-type atrophy	57 (30.1)	62 (38.0)	
Reflux esophagitis, *n* (%)	42 (22.2)	47 (28.8)	0.16
Barrett’s esophagus, *n* (%)	40 (21.1)	40 (24.5)	0.45
Bile-colored gastric juice, *n* (%)	31 (16.4)	22 (13.4)	0.45
*H. pylori* infection, *n* (%)			
Positive	36 (19.0)	6 (3.6)	
Negative			
Without a history of eradication	100 (52.9)	53 (32.5)	
After eradication	53 (28.0)	104 (63.8)	<0.01

IQR, interquartile range; BMI, body mass index; VG, verrucous gastritis.

#### Multivariate analysis

The multivariate analysis showed a significant association of gastric mucosal atrophy and *H. pylori* status with numerous VG. For gastric atrophy, both closed- (OR 9.29, 95% CI 4.04–21.37, *P *<* *0.001) and open-type atrophies (OR 8.10, 95% CI 3.41–19.24, *P *<* *0.001) were significantly associated with numerous VG. The OR for numerous VG was significantly higher in patients with the *H. pylori*-negative status than those with the *H. pylori*-positive status among patients with (OR 11.50, 95% CI 4.40–30.02, *P *<* *0.001) and without (OR 8.27, 95% CI 2.95–23.23, *P *<* *0.001) a history of eradication therapy. In particular, the OR associated with negativity after eradication/positive was 11.50, which was notably high (Table 4).

**Table 4.** Multivariate logistic-regression analysis for patient characteristics between numerous VG and classical VG groups

Characteristic	OR	95% CI	*P*-value
Age (years)	1.00	0.97–1.03	0.98
Male gender	1.01	0.59–1.71	0.98
BMI (kg/m^2^)	1.05	0.97–1.13	0.24
Total cholesterol (mmol/L)	1.00	1.00–1.01	0.27
Albumin (g/L)	1.08	0.57–2.04	0.81
Gastric mucosal atrophy			
No atrophy	1.00		
Closed-type atrophy	9.29	4.04–21.37	<0.001
Open-type atrophy	8.10	3.41–19.24	<0.001
Reflux esophagitis	1.61	0.86–2.99	0.13
Barrett’s esophagus	0.93	0.50–1.71	0.81
Bile-colored gastric juice	0.50	0.25–0.99	0.05
*H. pylori* infection			
Positive	1.00		
Negative			
Without a history of eradication	8.27	2.95–23.23	<0.001
After eradication	11.50	4.40–30.02	<0.001

VG, verrucous gastritis; BMI, body mass index; OR, odds ratio; CI, confidence interval.

### Follow-up and cancer outcomes

Of 621 patients, 305 (49.1%) underwent follow-up endoscopy until the end of 2018 and, of 180 *H. pylori-*positive patients, 136 (75.6%) had eradication therapy after endoscopy. Only one patient developed gastric cancer 2 years after endoscopy. He was 68 years old and had classical VG at this study period. His BMI was 26.5 and he had *H. pylori* infection with open-type atrophy. He did not receive eradication therapy. His cancer was a 0-IIa-type intramucosal, well-differentiated adenocarcinoma (<1 cm) of the antrum, which was successfully removed by endoscopic submucosal dissection. Endoscopic images were reviewed, but it was difficult to determine whether his cancer developed from previous verrucous lesions.

Two patients died of other cancers (hilar cholangiocarcinoma and sigmoid colon cancer) and two others died of acute-on-chronic liver failure and thrombotic microangiopathy.

## Discussion

This study retrospectively investigated the clinical and endoscopic features of VG, especially in relation to *H. pylori* infection and nutritional status. The results showed that VG was negatively associated with *H. pylori* infection but was positively associated with BMI and reflux esophagitis.

VG, also known as endoscopic erosive gastritis, complete erosion, varioliform erosions, and, in Japan, ‘octopus’ sucker’ (due to the lesions’ resemblance to octopus suckers, which are linearly arranged similar to that of an octopus’s limb), is a well-known lesion of the antrum that is occasionally observed during routine endoscopy. It is generally considered a benign lesion in the antrum and is unrelated to *H. pylori* infection, but there are no definite identification criteria for VG. Our study attempted to define and classify VG using chromoendoscopy. Although notably high and reddish verrucous erosions are easily noticeable, even with white light, verrucous lesions do not always appear that way. Chromoendoscopy aids in the accurate evaluation of the quantity and distributions of lesions.

Previous studies have shown similar or differing results compared to those of our study. A positive relationship was reported between gastroduodenal ulcers, especially duodenal ulcer, and erosive gastritis in the antrum in 1970s [1, 2, 10]. During that time, *H. pylori* was unknown, but we speculated that many patients with gastroduodenal ulcers included in the studies of Kawai, Green, and Sata [1, 2, 10] had *H. pylori* infection. In contrast, our study showed a negative relationship between VG and *H. pylori* infection. Gastric acid was considered the predominant factor in the genesis of erosive gastritis [2, 11] and, recently, antral erosion was reported to predict hyperchlorhydria in *H. pylori* negativity [12]. Obesity and high BMI were also associated with gastric erosions [3, 4, 13, 14]. Bile reflux was also thought to be a cause of reactive gastritis [15]; in recent years, bile acid [14] was thought to contribute to reflux esophagitis and Barrett’s metaplasia with related carcinogenesis [16]. However, in our study, bile-colored gastric juice was unrelated to VG, although it is uncertain whether bile-colored gastric juice accurately represents the extent of bile reflux.

The pylorus and distal antrum are anatomically important portions of the stomach and have a special role in gastric emptying and digestion. Pyloric closure occurs both by the contractions of the proximal muscle loop located 2–3 cm upstream from the pyloric ring on the greater curvature and the distal sphincter loop surrounding the pyloric ring and by the projection of longitudinal mucosal folds into the lumen. Some mucosal prolapse through the pyloric ring is considered a physiological phenomenon. Food and acid are known to increase pyloric tone and flow resistance [17]. Tiny elevated lesions of VG, especially the classical type, according to our definition, lie on the longitudinal mucosal fold of the terminal antrum. As noted above, our data showed that VG was positively related to BMI and reflux esophagitis. Nutrients and oily foods are related to high BMI [18] and patients with reflux esophagitis generally have a high gastric-acid concentration [18, 19]. These results suggest that mechanical overload due to eating and high acid concentration may be associated with VG. Other nutritional parameters, such as serum albumin as a protein reserve and total cholesterol as an abundance of calories, were not significant factors.

Our data also demonstrated a negative relationship between VG and *H. pylori* infection. In *H. pylori-*positive subjects with predominant antral gastritis, acid secretion increases whereas, in those with predominant body gastritis, acid secretion deceases [20, 21]. In our study groups, half of the non-VG group had *H. pylori* infection and 42.4% of them had open-type atrophy, suggesting that many non-VG patients had hypochlorhydria. *H. pylori* infection influences not only gastric-acid secretion, but also numerous vital pathways of the human systems [22]. Inflammatory cytokines also influence various cells to modulate gastric-acid production [23] and reduce gastric motility via the vagus nerve. *H. pylori* infection affects gut hormones, such as ghrelin, leptin, and glucagon-like peptide-1, which reduce appetite and gastric motility [24, 25], and changes in their serum concentration, up-regulation of acid secretion, and improvement of gastric motility after eradication have been reported [26, 27].

Our study comprised many patients with *H. pylori* negativity after eradication. Many of them tended to have numerous verrucous erosions compared to *H. pylori*-negative patients without a history of eradication, but we speculate that their acid concentrations and stomach motility levels were not higher than those of the latter. VG is supposed to occur only in the mucosa of the pyloric glands. Histologically, foveolae occupy the upper half and pyloric glands occupy the lower half of the pyloric gland mucosa. In contrast, foveolae occupy only one-fourth of the mucosal thickness in the mucosa of the oxyntic glands [28]. Pits are much deeper in the pyloric gland mucosa than they are in the oxyntic gland mucosa. Pyloric glands are shorter and less densely packed than oxyntic glands [29]. When strong terminal antral contraction occurs, the sparsely packed pyloric gland mucosa moves and prolapses more easily compared with the densely packed oxyntic gland mucosa. Histological characteristics of VG reported in the 1970s were foveolar hyperplasia, pyloric gland hyperplasia, connective-tissue proliferation, and thickening of the muscularis mucosae [1]. We noted similar findings and, occasionally, fibromuscular proliferation in the mucosal prolapsed syndrome of the rectum [30]. After eradication therapy, acute inflammation associated with chronic *H. pylori* gastritis improves and, coupled with other acid, hormonal, and neural changes, contraction of the pylorus becomes active. After long-standing inflammation, the antral–body transitional zone extends proximally with the replacement of normal body-type glands by irregular antral-type glands (pyloric metaplasia). The pyloric gland areas of patients with *H. pylori* negativity after eradication are supposed to be broader than those of *H. pylori*-negative patients without a history of eradication. The broad and sparsely packed pyloric gland mucosa or atrophic oxyntic gland mucosa might move and prolapse easily with strong terminal antral contractions, supposedly resulting in numerous verrucous lesions.

Regarding the relationship between VG and gastric cancer, Hirota *et al.* [31] reported that 1.37% of early-stage gastric cancer was suspected to develop from VG on analysis of precancerous lesions of 2,400 early gastric-cancer cases in Japan. The most common precancerous lesion was chronic atrophic gastritis (94.8%) and the frequency of VG as a precancerous lesion was low. In this study, only one patient developed early-stage gastric cancer. He had VG, but he also had chronic atrophic gastritis. The endoscopic features of VG sometimes resemble 0-IIa+IIc-type early-stage gastric cancer and the need for differential diagnosis is described in Japan [32]. Recently, there were two reports that linked VG to early-stage gastric cancer [7, 8]. In our study, we did not evaluate the histology of verrucous lesions. We often find intestinal metaplasia in biopsies of verrucous lesions, but we rarely find dysplasia or intraepithelial neoplasia in daily clinical practice. The aforementioned studies and ours were all retrospective studies. Therefore, large prospective studies are needed to evaluate the VG as a premalignant lesion.

Although the BMI of the VG group was higher than that of the non-VG group, its median value was 22.6 kg/m^2^, which is not indicative of obesity. Moreover, other nutritional indicators such as serum cholesterol were not significantly different between both groups. In addition, many patients in the VG group were *H. pylori*-negative, which might reflect a healthy stomach with good motility. Therefore, verrucous antral gastritis may not be a pathological phenomenon; instead, it may be a physiological phenomenon of a healthy stomach.

Several limitations of this study should be acknowledged. First, gastric motility was not measured and, second, histological assessments of both classical and numerous VG were not performed.

In conclusion, VG of the antrum was negatively associated with *H. pylori* infection but was positively related to BMI and reflux esophagitis. Furthermore, the number and distributions of verrucous lesions may depend on the extension of the pyloric gland area.

## Authors’ contributions

N.T. and Y.U. designed the study; acquired, analysed, and interpreted the data; and prepared the manuscript. N.T., Y.U., M.T., and Y.M. performed the endoscopic procedure, evaluated the subjects, and acquired the data. T.W., N.N., and M.K. provided advice regarding the design of the study and reviewed the final manuscript draft. All authors read and approved the final manuscript.
